# Cycles, waves, and pulses: Retinoic acid and the organization of spermatogenesis

**DOI:** 10.1111/andr.12722

**Published:** 2019-11-20

**Authors:** Rachel Gewiss, Traci Topping, Michael D. Griswold

**Affiliations:** ^1^ School of Molecular Biosciences and Center for Reproductive Biology Washington State University Pullman WA USA

**Keywords:** cycle, retinoic acid, synchronization, wave

## Abstract

**Background:**

Spermatogenesis in mammals is organized in a manner that maximizes sperm production. The central aspect of this organization is the cycle of the seminiferous epithelium that is characterized by an asynchronous repeating series of germ cell associations. These cell associations are the result of a fixed point of entry into the cycle at regular short time intervals and the longer time required for cells to fully differentiate and exit the cycle.

**Objective:**

This review will examine the current information on the action and metabolism of retinoic acid in the testis, the interaction of retinoic acid (RA) with the cycle and the spermatogenic wave, and the mechanisms that can lead to synchronous spermatogenesis. Finally, the unique applications of synchronous spermatogenesis to the study of the cycle and the mass isolation of specific germ cell populations are described.

**Materials and methods:**

Retinoic acid metabolism and spermatogonial differentiation have been examined by gene deletions, immunocytochemistry, chemical inhibitors, and mass spectrometry.

**Results, discussion, and conclusion:**

Both the Sertoli cells and the germ cells have the capacity to synthesize retinoic acid from retinol and in the mouse the entry into the cycle of the seminiferous epithelium, and the subsequent conversion of undifferentiated spermatogonia into differentiating spermatogonia is governed by a peak of RA synthesis occurring at stages VIII‐IX of the cycle. Normal asynchronous spermatogenesis can be modified by altering RA levels, and as a result the entire testis will consist of a few closely related stages of the cycle.

## 1 THE CYCLE, THE WAVE, AND RETINOIC ACID

**Figure 1:**
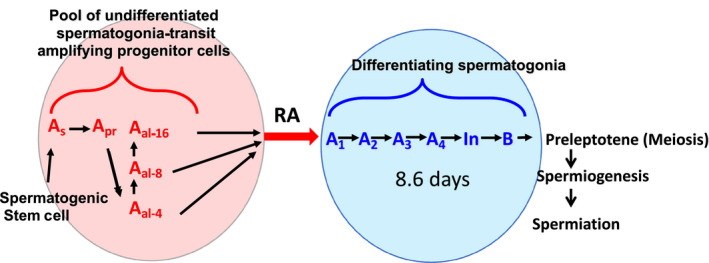
The effect of retinoic acid on the development of male germ cells. The so‐called ‘undifferentiated’ A spermatogonia originate as A single (A_s_) cells and undergo mitotic division with incomplete cytokinesis to form two A paired (A_pr_) cells. Further mitotic divisions with incomplete separation form syncytia of A aligned (A_al_) cells in chains of 4, 8, and 16 cells (red circle). The undifferentiated spermatogonia that are not self‐renewing spermatogenic stem cells are capable of undergoing the A to A1 transition (spermatogonial differentiation) in response to an RA pulse (red arrow). This differentiation event triggers development through the subtypes of differentiating spermatogonia (A1, A2, A3, A4, Intermediate, and B) until cells develop into pre‐leptotene spermatocytes prepared to enter meiosis (blue circle). At a given point along a seminiferous tubule, a new cohort of undifferentiated spermatogonia will undergo spermatogonial differentiation every 8.6 days in the mouse

The cycle of the seminiferous epithelium, described by Regaud^1^ and later in detail by Leblond and Clermont, is a fundamental characteristic of spermatogenesis in mammals.^1^, ^2^ The entry into the cycle occurs when ‘undifferentiated’ spermatogonia also known as ‘progenitor’ cells that proliferate from stem cells are simultaneously triggered to enter into a differentiation pathway.^3^ Recent studies in mice have shown this simultaneous entrance of A spermatogonia into the differentiation pathway requires retinoic acid (RA), the active form of vitamin A.^3^ It is clear from both genetic studies and the use of chemical inhibitors that the undifferentiated A spermatogonia in mice cannot enter the differentiation pathway to form A1, A2, A3, A4, Intermediate, and B spermatogonia without the action of RA (Figure 1). The time for a cell to complete differentiation from spermatogonia into spermatozoa is fixed for each species and appears to be invariable. Leblond and Clermont first deduced that ‘for a given species, each step of spermatogenesis has a constant duration,thus germ cell differentiation unfolds as if regulated by a rigid time‐scaled program’. There are no known mutants in mammals that can alter the length of the time of the cycle of the seminiferous epithelium. Franca et al, using germ cell transplantation put mouse germ cells into a rat testis and showed that the germ cell genotype specified the length of the cycle.^4^ The cell associations in each stage of the cycle of a species are a result of the time for a cell to enter the differentiation pathway and mature into a spermatozoan (usually 40+ days) and the frequency of recurring entrance of new cohorts of undifferentiated spermatogonia into the pathway (usually 8 to 14 days).

The spermatogenic wave initially described in detail by Perey et al was defined as a series of adjacent tubule segments that included all 14 possible cell associations comprising the stages of the cycle in the rat.^5^ A segment is the portion of a tubule occupied by one type of cellular association. Each seminiferous tubule has an overall U‐shape with both ends opening into the rete and if traced from the rete, each segment is followed by a segment with the next lowest number (known as the descent of the segmental order). This observation suggested that the rete testis or other segments can affect the stage of the adjacent segments. The descent of the segmental order is observed from both openings of the tubule into the rete testis and continues distally until there is a site of reversal of the segmental order.

## 2 METABOLISM OF RETINOIC ACID IN THE TESTIS

**Figure 2:**
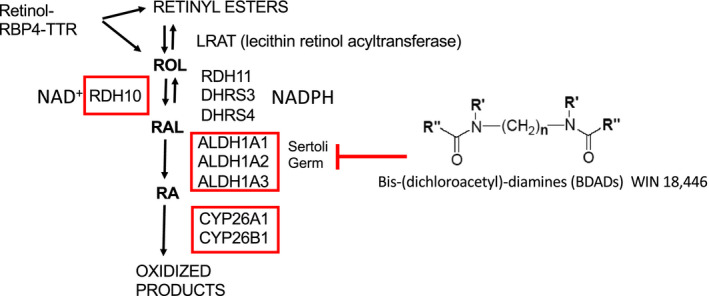
Retinoic acid metabolism in the testis. Retinol (Vitamin A) transits to the testis in a complex with retinol binding protein 4 (RBP4) and transthyretin. There lecithin retinol acyltransferase (LRAT) can convert retinol (ROL) into retinyl esters, or ROL can be converted into retinal (RAL). The ROL to RAL conversion is catalyzed by RDH10 in an NAD^+^‐dependent manner and is the rate‐limiting step in the conversion of ROL to RA. The reverse reaction can be carried out by one of several retinal reductases (RDH11, DHRS3, and DHRS4) and is dependent on NADPH. The second and irreversible step converts RAL to RA and is catalyzed in both Sertoli and germ cells by the aldehyde dehydrogenase 1A (ALDH1A) family of enzymes. This step can be blocked with the BDAD compound WIN 18,446, resulting in an arrest at the A to A1 transition that mimics *Rdh10* or *Aldh1a1‐3* genetic knockouts in Sertoli and/or germ cells. RA in the testis is degraded into inactive oxidized products by CYP26A1 and CYP26B1. The genes for each of the enzymes enclosed by the red boxes have been deleted in multiple cell types in the testis, and the results are discussed in the text

The half‐life of RA in the mouse testis was shown to be about 1.3 h^6^ The RA signaling system in the testis appears to be limited by the localized concentration of the ligand (ie, the synthesis and degradation of RA) rather than the receptors. In the enzymatic pathway for RA metabolism in the testis, retinol undergoes two oxidation steps to form RA which can activate available receptors and then is quickly oxidized to inactive metabolites (Figure 2). Retinol is delivered to the testis via retinol binding protein 4 (RBP4) in a complex with transthyretin.^7^ It has been shown that STRA6 can serve as a receptor for this delivery complex and may make the cellular uptake of RA more efficient. Sertoli cells and germ cells can esterify and store retinol or metabolize it to RA.^8^ The first oxidation step to convert RA to retinal is reversible and the oxidative enzyme that plays a major role in the first round of spermatogenesis is retinol dehydrogenase 10.^9^ This enzyme requires NAD+, while the retinal reductases that catalyze the reverse reaction (RDH11, DHRS3, and DHRS4) require NADPH.^10^ The rate‐limiting step in the synthesis of RA is this first oxidative reaction.^11^ The second oxidative step to convert retinal to RA can be carried out by three retinaldehyde dehydrogenases (ALDH1A1, ALDH1A2, and ALDH1A3). There is very little ALDH1A3 in the mouse testis, and its significance is unclear.^6^ RA is oxidized by two P450 enzymes (CYP26A1 and CYP26B1) into presumably inactive metabolites. The first round of spermatogenesis is blocked at the undifferentiated A spermatogonia stage when the *Rdh10* gene or the three *Aldh1a* genes are knocked out in Sertoli cells.^9^, ^12^ However, if the mutants with blocked A spermatogonia are given an exogenous RA injection, the A to A1 spermatogonial transition occurs and spermatogenesis continues through many cycles.^9^, ^12^, ^13^ Thus, it is clear that the first round of germ cell development is regulated by RA generated primarily in Sertoli cells, but once germ cells more advanced than spermatogonia are formed they become a source of RA sufficient to drive spermatogenesis in the normal cyclic manner. This concept was elegantly confirmed when the three *Aldh1a* genes were knocked out in both Sertoli cells and germ cells.^12^ The transition of A to A1 spermatogonia was blocked but the injection of exogenous RA allowed only one cohort of A undifferentiated spermatogonia to proceed through spermatogenesis and did not support continuous spermatogenesis. A similar genetic dependency on both Sertoli cells and germ cells was seen in the analysis of *Cyp26a1* and *Cyp26b1* expression.^14^ The genetic knockout of *Cyp26a1* in one cell type or in both cell types had no effect on fertility. However, while the knockout of *Cyp26b1* in either Sertoli cells or germ cells also did not affect fertility, the deletion of this gene in both Sertoli cells and germ cells resulted in smaller testes and infertility with noticeable morphological abnormalities.

While some of the enzymatic parameters of RA synthesis in the seminiferous tubules have been revealed, very little is known about how RA synthesis is initiated so that spermatogenesis occurs in asynchronous continuous cycles. A transgenic mouse model expressing beta‐galactosidase under the control of an RA response element was used to examine the onset of the cycle in post‐natal mice.^15^^,^ 16 ^,^ 17 Active RA signaling in these mice resulted in the synthesis of beta‐galactosidase that was visualized histologically. Beta‐galactosidase activity was found to be distributed non‐uniformly in a patch‐like manner along the seminiferous tubules, suggestive of the start of the asynchronous cycle. Additionally, beta‐galactosidase activity in premeiotic germ cells colocalized with STRA8 protein and was induced in most germ cells with exogenous RA treatment, indicating that the rate‐limiting factor was the presence of the RA ligand.

**Figure 3:**
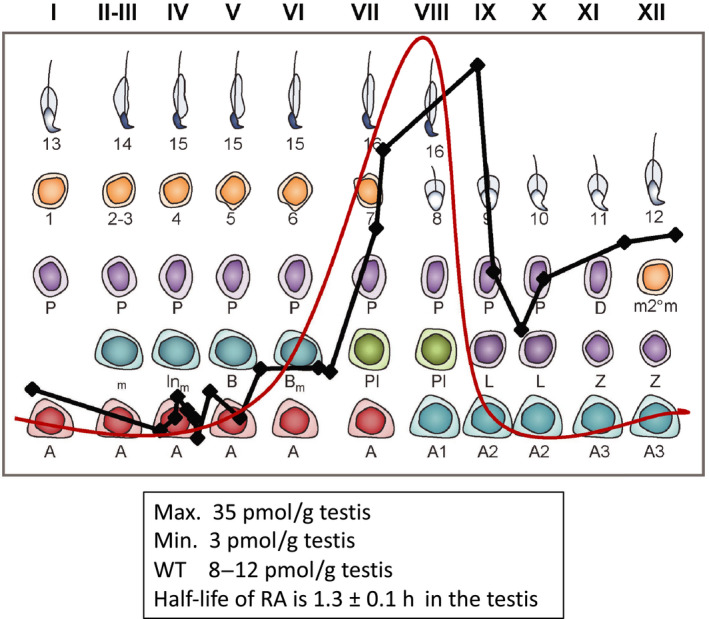
Retinoic acid pulse across the cycle of the seminiferous epithelium. Red line shows the predicted RA pulse based on previous data, black line shows actual, relative RA values across the cycle in stage‐synchronized testes (data from.^21^ RA levels peak at late stage VIII/early stage IX and reach a maximum value of 35 picomoles per gram testis (pmoles/g testis). Minimum RA values around 3 pmoles/g testis were observed in stages II through V. In unsynchronized adult testes where all stages of the seminiferous epithelium are present, RA values averaged around 8‐12 pmoles/g testis

The bis‐(dichloroacetyl)‐diamines (BDAD) compounds inhibit aldehyde dehydrogenases and are very effective inhibitors of testicular ALDH1A enzymes (Figure 2).^18^ The BDAD that has been most commonly used in studies on spermatogenesis is WIN 18,446. Treatment of mice with WIN 18,446 prevents the synthesis of retinoic acid and blocks the progression of undifferentiated A spermatogonia into the differentiation pathway. When neonatal mice are treated with this compound over time (7‐8 days), the A undifferentiated or progenitor spermatogonia accumulate but do not differentiate. If the mice are then treated with RA, the block is released and essentially all progenitor spermatogonia quickly become A1 and continue through spermatogonial development. If these treated mice are raised to adulthood, they are fertile but the entire testis is synchronized to a few related stages of the cycle of the seminiferous epithelium.^19^^,^ 20 The timing of the cycle remains intact but there is no spermatogenic wave. By monitoring the time after the injection of RA, the testes can be synchronized to all related stages of the cycle. We then used mass spectrometry to quantify the amount of RA present in the testes that were synchronized to different stages of the cycle.^21^ The results (summarized in Figure 3) show that there is a pulse of RA appearing across stage VIII and stage IX of the cycle. The peak encompasses the stages where the transition of A to A1 spermatogonia occurs and represents the initiation point of the cycle.

In their early descriptions of the cycle of the seminiferous epithelium, Leblond and Clermont speculated that ‘some other factor(s) must be involved to account for the simultaneous entrance in spermatogenesis of spermatogonial stem cells’.^2^ In addition, in the depiction of the wave as an ordered association of consecutive segments, Regaud^1^ suggested that the spermatogenic wave could be explained by an ‘impulse’ that traveled along the tubules and that the wave is in space what the cycle is in time.^1^ The stem cells actually give rise to progenitor undifferentiated spermatogonia that form aligned syncytia of 2 to 16 cells and enter into the differentiation pathway and the cycle simultaneously (see Figure 1). RA clearly could explain the notion of ‘some other factor’ proposed by Clermont, but does it fit the description of an ‘impulse that travels along the tubules’? The organization of numerically related segments such as occurs in the descent of the segmental order would suggest that segments interact. However, recent 3‐dimensional reconstructions of tubule architecture and segment arrangement suggest a far more complex relationship between segments.^22^^,^ 23 Perey noted in 1961 that many waves were interrupted by irregularities in the segmental order that he termed modulations. Both Perey et al and recently, Nakata et al, showed that about 80% of the waves were interrupted by modulations.^5^, ^22^, ^23^ In the 3‐dimensional reconstruction of the nine tubules found in an adult mouse testis, Nakata et al showed that many tubules had branch points, some as many as 5 or 6 branches to tubules with a blind end.^22^^,^ 23 He also identified 71 waves as defined by Perey et al and 19 sites of reversal of the segmental order. Perey et al stated that, ‘The variability in the features of the wave and the presence of modulations made it unlikely that the wave is triggered by an impulse progressing along the tubule, as had been suggested by Regaud’.^5^ In addition, because of these variations in the wave and the extreme consistency in the cycle, Perey stated that the wave is not in space what the cycle is in time.^5^ The statements by Perey et al seem to be reinforced by the results of Nakata et al The fact that testes can be synchronized to nearly a single stage and yet maintain the overall normal timing of the cycle would indicate that the generation of an RA pulse is likely segment autonomous. In regions of the tubule where the segments are in sequential order, the pulse could arise autonomously in stages VIII and IX and would appear to (though not actually) move along the tubule. How and why the descent of the segmental order from the rete occurs and how one segment might influence another remains an enigma. The segment autonomous nature of the generation of the pulse could also explain how the cycle could be generated in humans and higher primates where the organization of the segments is not linear along the tubule.

## 3 APPLICATIONS OF TESTICULAR SYNCHRONY

**Figure 4:**
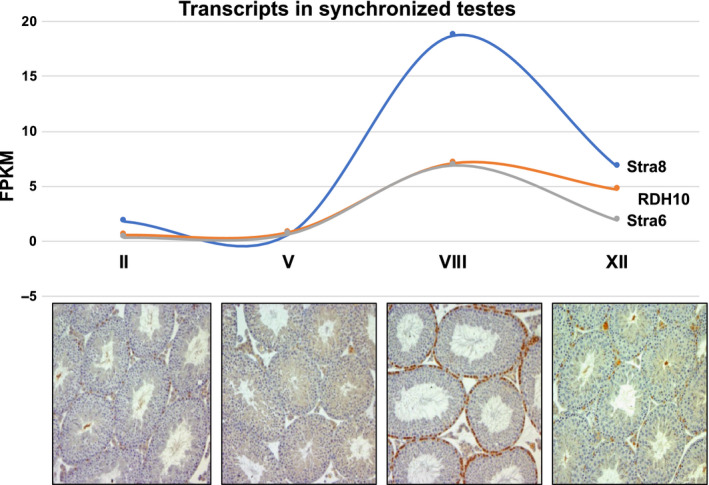
Retinoic acid‐responsive gene transcripts across the cycle of the seminiferous epithelium. *Stra8* (blue line) showed the greatest transcriptional activity in response to RA signaling, but *Rdh10* (orange line) and *Stra6* (gray line) transcript levels also directly correlate with the RA pulse. Cross sections for each of the represented stages (II, V, VIII, and XII) of stage‐synchronized testes are shown below the transcript data. Brown‐stained cells at the basement membrane represent STRA8‐positive spermatogonia and pre‐leptotene spermatocytes at the A to A1 transition (Stage VIII)

The ability to use the information that has been obtained on RA and the cycle to synchronize testes so that they only contain a few related stages has been extremely useful in a number of studies. When WIN 18,446 was used as described above to synchronize pre‐pubertal mice that were allowed to become adults, testes could be isolated that contained only 1 or 2 stages, and if collected at different times synchronized testes covering each part of the cycle could be obtained. That technique allowed us to obtain the RA measurements across the different stages as described above and generate RNA‐seq data on the whole testis at each stage (Figure 4).^20^ If the synchronization is applied to testes where there is a fluorescent tag on germ cells it is possible to isolate abundant, highly purified germ cells at essentially every stage of development. Chen et al used this technique and combined it with single‐cell RNA‐sequencing to obtain the transcriptome of the germ cells throughout development from A1 spermatogonia to elongating spermatids.^24^ We have recently used this technique to obtain transcriptome information on the undifferentiated A and differentiating A1, A2, A3, A4, Intermediate, and B spermatogonia (Testis Workshop 2019 abstract 30). The Page Laboratory published the details of this technique^25^ and used it to examine the role of STRA8 in WT and KO pre‐leptotene spermatocytes.^26^ While the synchronization technique is extremely useful and has allowed studies that were not possible previously, it should be noted that the testes only remain synchronized within a given time window. The WIN 18 446 synchronization procedure can result in tight testicular synchrony from 9 dpp mice (time of RA injection) through the early adult. Analyses of WIN 18,446 synchronized testes at 90‐days and 180‐days post‐injection revealed that testes were no longer showing synchronous spermatogenesis, but WIN 18,446/RA treatment of neonatal mice did result in long‐lasting alterations of the arrangement of spermatogenesis within the testis.^20^ At both time points instead of the normal arrangement of 12 stages observed within cross‐sections of control testes, the stages were arranged in a sequential fashion along the dorsal‐ventral axis. Mating tests of WIN 18,446/RA‐treated animals and of their offspring revealed these animals were fertile and produced normal litter sizes, indicating that the spermatozoa produced after the cessation of WIN 18,446 treatment were perfectly functional. While the development of the RA pulse and the entry into spermatogenesis appears to be segment‐independent, there is clearly a developmental bias toward asynchrony. Factors driving that bias and how segments may interact remain to be investigated Table 1).

**Table 1:** Methods to generate synchronous spermatogenesis in mice

The key is to treat testes that contain primarily undifferentiated A spermatogonia with RA	
Vitamin A deficient	Rats maintained on a vitamin A deficient diet over many weeks contain primarily undifferentiated A spermatogonia.^27^
Neonate	Mice injected with RA on days 1‐4 post‐natal. Synchrony less well characterized than Win 18 446 method.^16^
Win 18,446 block	Mice treated for 2 to 9 days after birth with WIN 18,446 followed by a single injection of RA. Synchrony is well characterized.^19^^,^ 20

## 4 CONCLUSIONS AND FUTURE DIRECTIONS

The understanding of the role of retinoic acid in the generation of the cycle of the seminiferous epithelium in mammals and in the commitment of differentiating germ cells adds molecular details to the morphological descriptions initially made over a century ago. The cycle is clearly driven by a pulse or peak of RA synthesis coincident with the stages where spermatogonia enter the differentiation pathway and commit to meiosis. The synthesis of the RA signal is a result of metabolism in the Sertoli cells and in the germ cells. The synchronization of spermatogenesis so that only a few stages of the cycle are present in a rat testis was first shown in studies using rodents that were deprived of vitamin A in the diet.^27^ While this approach was useful, it was difficult since it took 12 to 14 weeks and resulted in animals with many health issues. The more recent studies have built on the dietary approach and found that synchronous spermatogenesis can be achieved in mice in other ways, using RA injection into neonates or administration of WIN 18 446. Synchrony after these treatments is simple, much quicker and has no visible effect on the health of the mice. The uses of this technique to isolate to purity any of the diverse germ cells found in the testis in abundance has already allowed for unique molecular studies, and will hopefully lead to a greater understanding of spermatogenesis and meiosis.
